# PSSP-RFE: Accurate Prediction of Protein Structural Class by Recursive Feature Extraction from PSI-BLAST Profile, Physical-Chemical Property and Functional Annotations

**DOI:** 10.1371/journal.pone.0092863

**Published:** 2014-03-27

**Authors:** Liqi Li, Xiang Cui, Sanjiu Yu, Yuan Zhang, Zhong Luo, Hua Yang, Yue Zhou, Xiaoqi Zheng

**Affiliations:** 1 Department of General Surgery, Xinqiao Hospital, Third Military Medical University, Chongqing, China; 2 Department of Orthopedics, Xinqiao Hospital, Third Military Medical University, Chongqing, China; 3 Institute of Cardiovascular Diseases of PLA, Xinqiao Hospital, Third Military Medical University, Chongqing, China; 4 Key Laboratory of Biorheological Science and Technology, Ministry of Education, College of Bioengineering, Chongqing University, Chongqing, China; 5 Department of Surgery, The University of Michigan Medical School, Ann Arbor, Michigan, United States of America; 6 Department of Mathematics, Shanghai Normal University, Shanghai, China; 7 Department of Biostatistics and Computational Biology, Harvard School of Public Health, Boston, United States of America; National Institute of Genomic Medicine, Mexico

## Abstract

Protein structure prediction is critical to functional annotation of the massively accumulated biological sequences, which prompts an imperative need for the development of high-throughput technologies. As a first and key step in protein structure prediction, protein structural class prediction becomes an increasingly challenging task. Amongst most homological-based approaches, the accuracies of protein structural class prediction are sufficiently high for high similarity datasets, but still far from being satisfactory for low similarity datasets, i.e., below 40% in pairwise sequence similarity. Therefore, we present a novel method for accurate and reliable protein structural class prediction for both high and low similarity datasets. This method is based on Support Vector Machine (SVM) in conjunction with integrated features from position-specific score matrix (PSSM), PROFEAT and Gene Ontology (GO). A feature selection approach, SVM-RFE, is also used to rank the integrated feature vectors through recursively removing the feature with the lowest ranking score. The definitive top features selected by SVM-RFE are input into the SVM engines to predict the structural class of a query protein. To validate our method, jackknife tests were applied to seven widely used benchmark datasets, reaching overall accuracies between 84.61% and 99.79%, which are significantly higher than those achieved by state-of-the-art tools. These results suggest that our method could serve as an accurate and cost-effective alternative to existing methods in protein structural classification, especially for low similarity datasets.

## Introduction

As the basic compositions of life, proteins play a central role in most cellular functions such as gene regulation, metabolism and cell proliferation. In order to interpret the function of a new protein sequence, it is fundamental to understand its 3D structure. Since the knowledge of protein structural class provides useful information towards the determination of its 3D structure, prediction of protein structural class from sequence data becomes a hot topic in computational biology, especially with the development of high-throughput technologies [1]. Generally, proteins have irregular surfaces and complex 3D structures, but they are formed regularly in regional fold patterns at secondary structure level. Based on the contents of their secondary structures, known protein structures are classified into four categories, all-α, all-β, α/β and α+β. All-α and all-β proteins consist of only α-helices and β-strands, respectively. The α/β and α+β proteins are mixed with α-helices and β-strands, where the former consist of parallel β-proteins and the latter anti-parallel β-proteins. Experimental approaches to determining the structure information of a protein, including X-ray Diffraction and Nuclear Magnetic Resonance, are costly and time-consuming, and thus not capable of completely meeting researchers' demands. Therefore, high-throughput computational approaches are brought to the forefront of this issue.

As a typical pattern recognition problem, computational methods for protein structural class prediction consist of three main steps: i) protein feature representation; ii) algorithm selection for classification; iii) optimal feature selection. Among the three steps, feature extraction is the most critical factor for the success of protein structural class prediction. For this step, models in common use include amino acid composition (AAC), polypeptide composition, functional domain composition, physicochemical features [2], PSI-BLAST profiles [3] and function annotation information [4]. Despite some success in prediction tasks, a carefully engineered integrated feature model generally offers higher accuracy and stability than those with a single feature. From this basic point, information from PSI-BLAST profiles, PROFEAT and Gene Ontology is integrated into the principal features of our model.

However, initial feature vector inevitably contains noisy information and some redundancies, which could severely affect the prediction results. In order to highlight the actual informative features, a feature selection step is needed. Commonly adopted feature selection algorithms for classification problems include F-score, T-statistic, MIT correlation, 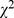-statistics and so on [5]. However, majority of these feature selection algorithms are based on the evaluation and ranking of individual features. Hence some weak features, which may have a strong combination effect but weak signal evaluated individually, could be neglected by these algorithms. Another group of feature selection tools, such as Correlation-based Feature Selection (CFS) [6] and Genetic Algorithm [7], could rank the values of features as subsets rather than individually. But they may fail to select locally predictive features, especially when these are overshadowed by strong and globally predictive ones. To overcome the shortcomings, SVM-RFE was proposed by ranking features based on the mutual information in the whole feature space [8]. SVM-RFE discretely removes only one feature from the whole feature vectors, and thus could take advantage of locally predictive features with relatively less computational cost.

In this work, we propose a novel computation method that combines SVM with PSI-BLAST profile, physical-chemical property and functional annotations to further improve the prediction of protein structural class. Here, a simple and powerful sequence representation model (PSSP-RFE) is employed to transform the original profile. The feature vector is then input to an SVM classifier to perform the prediction. Jackknife cross-validation tests on seven widely used benchmark datasets show that our method presents satisfying prediction accuracies in comparison with other existing methods.

## Materials and Methods

### 1. Datasets

Two groups of datasets were adopted to evaluate the proposed method. One is the high similarity datasets, including Z277 and Z498, which consist of 277 and 498 protein domains respectively. The other group consists of all low similarity datasets, i.e., 1189 [9], D640 [9], 25PDB [10], D8244 [11] and D1185 [11]. Pairwise sequence similarities in these datasets are all lower than 40%. The detailed information about the seven datasets is listed in **Table 1**.

**Table 1:** Seven benchmark datasets used to train and test our predictor.

Dataset	Number of proteins				
	all-α	all-β	α/β	α+β	total
1189	223	294	334	241	1092
D640	138	154	177	171	640
25PDB	443	443	346	441	1673
Z277	70	61	81	65	277
Z498	107	126	136	129	498
D1185	251	258	199	477	1185
D8244	1744	1929	2357	2214	8244

### 2. Linear predictive coding of the PSI-BLAST profiles

The PSSM of a protein sequence represents homolog information affiliated with its aligned sequences, where the 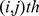 element is the score of the amino acid residue in the 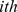 position being mutated to amino acid type 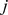 during the evolutionary processes. PSSM for each sequence was generated by the PSI-BLAST program against the NCBI's non-redundant (NR) database under the parameter setting 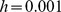 and 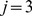. The PSSM elements are mapped to the range of [0,1] by the following standard sigmoid function,
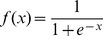
(1)where 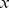 is the original PSSM value.

Next, the linear predictive coding (LPC) scheme [12], a tool widely used in speech recognition, was applied to optimally parameterize the signal. LPC is one of the most useful methods for encoding good quality speech at a low bit rate and provides extremely accurate estimates of speech parameters. The derived coefficients were used as quantitative features replacing signal intensities. Here, for each column of PSSM, we utilized LPC analysis process to extract 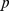 features. This allowed the transformation of each PSSM to a 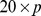 feature vector for each protein.

### 3. Gene function annotation features

GO term data are available from ftp://ftp.ebi.ac.uk/pub/databases/GO/goa/UNIPROT/(released on October 10, 2013). In the first step, all GO terms corresponding to the five datasets were searched for the protein entries. Note that current available GO terms did not cover all proteins, so the proteins without known GO terms were discarded from the datasets in the following analysis. Since the numbers (28, 34, 60, 3 and 14 for 1189, D640, 25PDB, Z277 and Z498) are quite small compared to the total number of sequences, this filtering step would not affect the final accuracy seriously. After this step, 3245, 2555, 4740, 941 and 931 different GO numbers are obtained for 1189, D640, 25PDB, Z277 and Z498, respectively. To further simplify the representation of proteins in all datasets, we created a vector to represent the GO terms as follows. Suppose 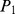 is a protein in 1189 dataset and it corresponds to 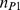 GO numbers. We first list all 3245 GO terms related to the entire dataset and formed a vector:
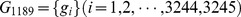
(2)where 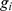 is the 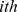 GO term. So 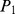 could be represented as a 3245-dimension vector, i.e.,
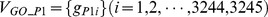
(3)where
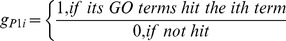
(4)


Following the above procedure, each protein of the five datasets could be represented as a feature vector, with the dimensions 3245, 2555, 4740, 941 and 931 for 1189, D640, 25PDB, Z277 and Z498, respectively. Here 1189 dataset was selected for optimization of the parameters in LIBSVM, and chosen to predict the structural class of a new protein. 1189 dataset is selected as the benchmark dataset due to its low pairwise sequence similarity, large population to ensure a high statistical power and wide adoptions in many published works.

### 4. Extracting structural and physicochemical features by PROFEAT

PROFEAT is a web server for computing the frequently used structural and physicochemical features of proteins and peptides from amino acid sequence [2]. These features include dipeptide composition, quasi-sequence-order descriptors, sequence-order-coupling number, and various structural and physicochemical properties. PROFEAT provided a satisfactory way to predict the structural, functional and interaction profiles of proteins and peptides irrespective of sequence similarity. In this study, by inputting a query protein sequence and selecting all the PROFEAT features, we finally acquired a 1080-dimension vector of PROFEAT feature for each query protein.

### 5. Feature extraction by SVM-RFE

With a limited number of training examples, a small amount of features often result in a better generalization of machine learning algorithms (Occam's razor) [13]. Meanwhile, the increased dimensions of the feature vectors would increase the amount of calculation of some machine learning methods, such as support vector machine and neural network. For this reason, an R script from SVM-RFE algorithm package [14] was introduced to select top features. Firstly, PSSM, PROFEAT and GO features of each protein were integrated into a feature vector. All the feature vectors of proteins for each dataset would be used to construct a feature matrix, where each column represents a feature and each row represents a sample. Then, training an SVM with a linear kernel, we ran the SVM-RFE algorithm to get a rank list of all features by removing only one feature with the smallest ranking criterion each time. The first item in the rank list is the most relevant to perform protein structural class prediction, and the last item has the least relevant feature. Finally, we were able to select different top 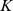
features according to the ranking list.

### 6. The SVM ensemble classifier

**Figure 1:**
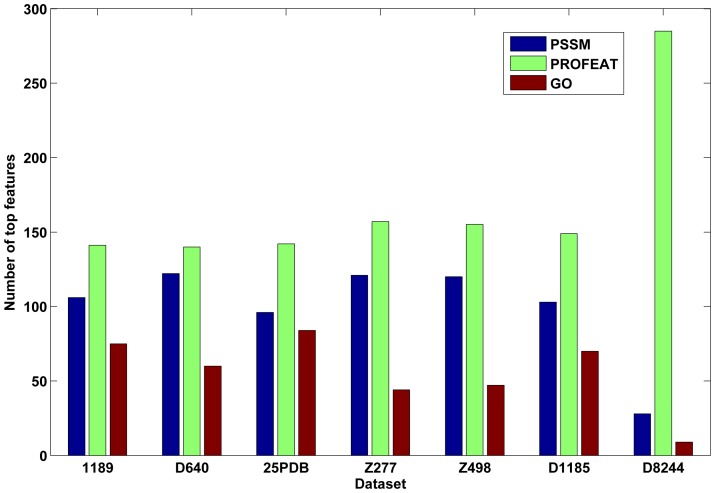
This graph shows the distribution of top322 features. SVM-REF ranked the features according to their ability to separate different categories for each dataset. So the ranking lists and top features are different for different datasets. Apparently, proportions of different kinds of features are consistent for all seven datasets, i.e., physical-chemical properties reflected by PROFEAT constitute the majority group, followed subsequently by PSSM and GO annotation features. The bar chart shows the numbers of three different kinds of features in top features for each dataset.

Support vector machine (SVM) is a supervised learning model that is popular in many pattern recognition problems including predictions of protein structural class, subcellular location, binding ligands, and identifying the functional roles of genes, etc. [15], [16]. Rather than the whole dataset, the SVM determines the margin between two classes based only on support vectors, which makes it less prone to overfitting than other classification methods [17]. Compared to other machine learning methods, SVM has the advantages of high performance, absence of local minima, the speed and ability to deal with multidimensional datasets with complex relationships among the data elements. As the type of kernel function decides the performance of SVM, we selected the most popular radial basis function (RBF) kernel for its better performance in different kinds of prediction tasks [4]. Here the LIBSVM software package was employed to enforce the SVM classifier. LIBSVM has two tunable parameters, i.e., the parameter 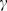 and regularization parameter 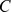, which could affect the accuracy of protein structural class prediction. In this article, the two parameters are also optimized based on the 1189 dataset by a grid search strategy. However, feature vectors optimized by different datasets may also have slight difference (**Fig. 1**).

Prediction of protein structural class is usually formulated as a multi-class classification problem. A simple way to deal with the multi-class classification is to reduce the multi-classification to a series of binary classifications. During this study, we adopted the *one-versus-one* method, i.e., 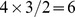 binary classification tasks were constructed for each dataset. Compared to the *one-versus-one* approach, the *one-versus-rest* strategy has the problem that the numbers of positive and negative training data points are not symmetric [18].

### 7. Assessment of prediction performances

In statistical prediction, jackknife test, independent dataset test and sub-sampling test are the most commonly used methods for evaluating the effectiveness of predictors. Due to its objectivity and rigidity, the jackknife test is more prevalent for examining the power of predictors than other cross-validation procedures [19], so it was adopted to validate our predictor. The accuracy, overall accuracy and Matthew's Correlation Coefficient (MCC) are formulated as follows:
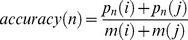
(5)

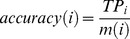
(6)

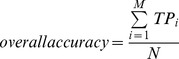
(7)

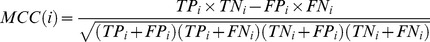
(8)


**Figure 2:**
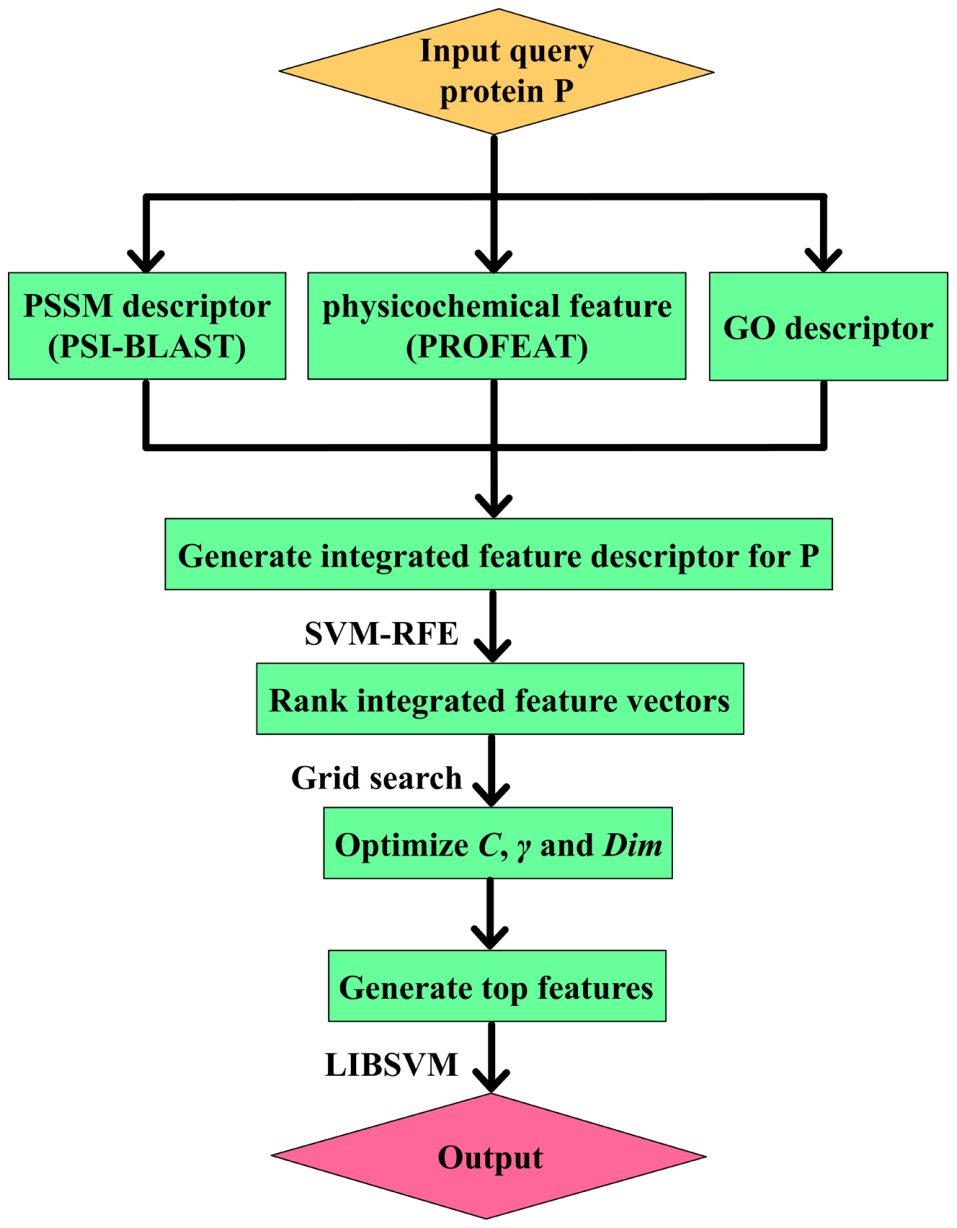
This graph shows the pipeline that goes from the query sequence to the final output as well as all intermediate steps.

Here, 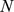 denotes the total number of proteins, 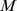 denotes the class number, 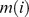 and 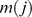 are the numbers of the proteins in classes 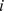 and 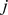; 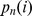 and 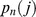 represent the numbers of the correctly predicted proteins of class 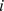 and class 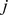 by binary classifier 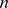. 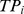, 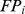, 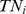 and 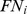 denote true positives, false positives, true negatives, and false negatives in class 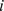, respectively. **Fig. 2** shows the pipeline that goes from the query sequence to the final output as well as all intermediate steps.

## Results and Discussion

### 1. Parameter selection

**Figure 3:**
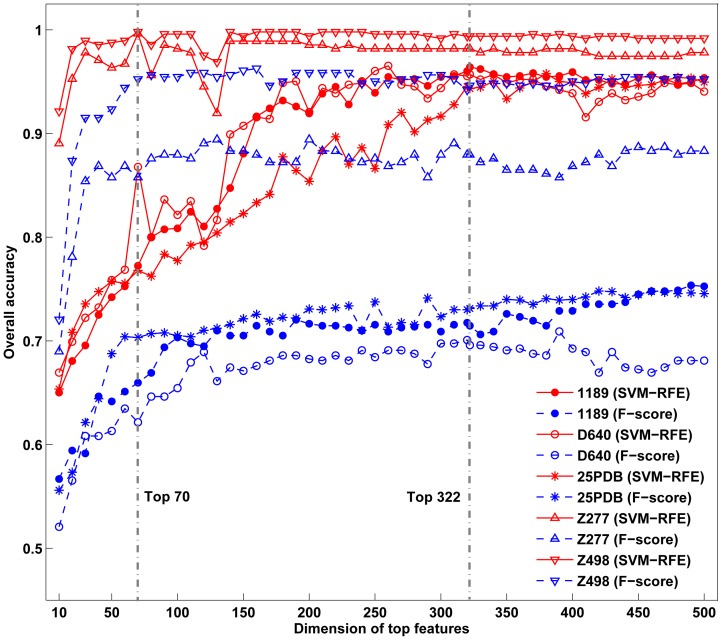
This graph shows comparison of prediction accuracies by SVM-RFE and F-score. Gray dotted lines highlight the selected top features for high (top 70) and low (top 322) similarity datasets.

In this study, we used a grid search strategy to select the parameters in LIBSVM, which depend on the dimension 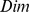 of the top feature vector of proteins. By combining the lpc3, lpc4, …, lpc9, lpc10, PROFEAT and GO features, we firstly obtained a 5365-dimension feature vector for each protein. Then we gave each feature a score based on SVM-RFE and ranked these by their importance. To further determine the optimal accuracy and corresponding dimensions, we calculated the accuracy at each dimension from top1 to top500, and found that the accuracy at top322 was the highest for 1189 dataset (**Fig. 3**), which was selected for optimizing the parameters in LIBSVM. Thus top322 features and the corresponding parameters (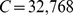, 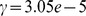 and 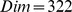) were selected to compute the accuracies for all three low similarity datasets. For two small datasets Z277 and Z498, a lower dimension top70 was adopted for their high accuracies and small sample sizes (**Table 2**). It should be noticed that parameters optimized from different datasets could be different, but they have significant overlap based on our result. For example, 117 common PROFEAT features are detected among the top322 features for datasets Z277 and Z498, given the p-value of 4.56×10^−21^ by the Fisher's exact test. Actually, we also tried feature vectors optimized by the other three datasets, and the corresponding predictions for all datasets are quite similar, which showed the robustness of our algorithm to the selection of feature vectors.

**Table 2:** Prediction performances on seven datasets by our method.

Dataset	all-α		all-β		α/β		α+β		Overall accuracy(%)
	accuracy(%)	MCC	accuracy(%)	MCC	accuracy(%)	MCC	accuracy(%)	MCC	
1189	94.88	0.9329	96.77	0.9585	96.59	0.9316	97.06	0.9141	96.40
D640	95.49	0.9329	96.55	0.9502	96.95	0.9225	93.87	0.9120	95.70
25PDB	94.90	0.9211	95.49	0.9220	95.83	0.9139	91.43	0.8675	94.34
Z277	100.00	1.0000	98.31	0.9892	100.00	0.9913	100.00	1.0000	99.64
Z498	100.00	0.9939	99.15	0.9944	100.00	0.9949	100.00	0.9947	99.79
D1185	83.06	0.7741	82.68	0.7821	79.79	0.7759	88.50	0.7790	84.61
D8244	86.98	0.8376	90.72	0.8444	93.52	0.8379	82.12	0.7554	88.42

### 2. Comparison with existing methods

**Figure 4:**
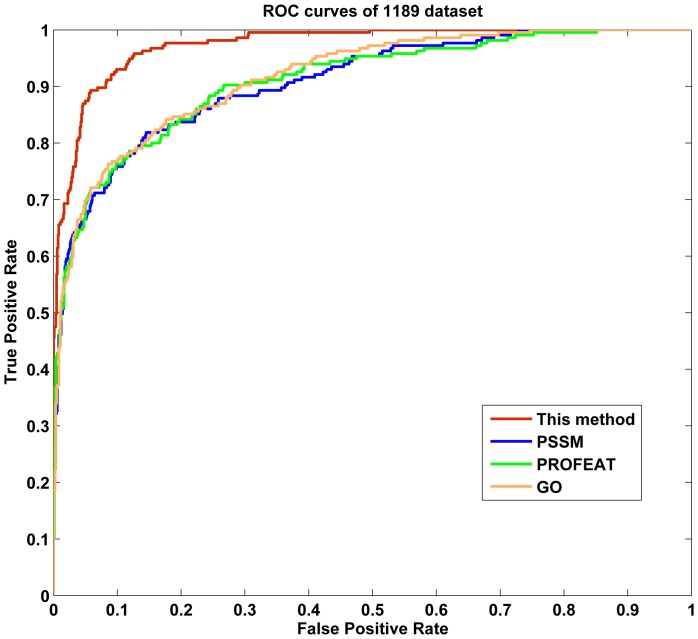
This graph shows the ROC curves of 1189 dataset.

We next compare our model with some previous methods based on the same datasets (**Tables 3** **–** **9**). As is shown, our method attained higher accuracies for low similarity datasets compared to previous methods. For instance, the overall accuracy of our method on 1189 dataset is 96.40%, higher than that by all other methods (from 12.9% to 42.5%). To illustrate the prediction performance of our method across different parameter settings, a receiver operating characteristic (ROC) curve was implemented. As we know, ROC curve is applicable to evaluate the prediction performance of a binary classifier, but structural class prediction is a four-class prediction problem. To deal with this problem, we first transformed structural class prediction to four binary classifiers using *one-versus-rest* strategy, and then averaged the four binary ROC curves as the final output of a method. **Fig. 4** shows the averaged ROC curves for 1189 dataset by our method and the other three approaches. We could observe that the area under curve (AUC) of our method is 0.9738, which is significantly higher than those by PSSM, PROFEAT and GO features individually (AUCs are 0.9085, 0.9099 and 0.9172, respectively). Similar results were obtained for the other six datasets (**Figs. S1–S6**). In addition, our method also obtained better prediction results on the other two low similarity datasets. For D640 dataset, our method achieved an accuracy of 95.70%, which was significantly higher than those achieved using methods listed in **Table 4** (from 12.26% to 33.40%). For 25PDB dataset, our method achieved an accuracy of 94.34% and also outperformed all other methods listed in **Table 5**. In addition, good performances were also obtained on two non-redundant datasets D1185 and D8244 (**Tables 6** **&** **7**).

**Table 3:** Performance comparison of different methods on 1189 dataset.

Method	Prediction accuracy (%)				
	All-α	All-β	α/β	α+β	Overall
Logistic regression by Kurgan and Homaeian (2006) [10]	57.0	62.9	64.7	25.3	53.9
Markov-SVM by Qin et al. (2012) [27]	53.8	79.3	68.3	32.0	60.3
IB1 by Chen et al. (2008) [28]	65.3	67.7	79.9	40.7	64.7
AAD-CGR by Yang et al. (2009) [29]	62.3	67.7	66.5	63.1	65.2
AADP-PSSM by Liu et al. (2010) [30]	69.1	83.7	85.6	35.7	70.7
AATP by Zhang et al. (2012) [31]	72.7	85.4	82.9	42.7	72.6
AAC-PSSM-AC by Liu et al. (2012) [23]	80.7	86.4	81.4	45.2	74.6
SVM by Ding et al. (2012) [32]	93.72	84.01	83.53	66.39	81.96
MODAS by Mizianty and Kurgan(2009) [33]	92.3	87.1	87.9	65.4	83.5
Our method	94.88	96.77	96.59	97.06	96.40

**Table 4:** Performance comparison of different methods on D640 dataset.

Method	Prediction accuracy (%)				
	All-α	All-β	α/β	α+β	Overall
SCEC by Chen et al. (2008) [28]	73.9	61.0	33.9	81.9	62.3
SCPRED by Kurgan et al. (2008) [34]	90.6	81.8	85.9	66.7	80.8
SVM by Ding et al. (2012) [32]	94.93	76.62	89.27	74.27	83.44
Our method	95.49	96.55	96.95	93.87	95.70

**Table 5:** Performance comparison of different methods on 25PDB dataset.

Method	Prediction accuracy (%)				
	All-α	All-β	α/β	α+β	Overall
Bagging with random tree by Dong et al. (2006) [35]	58.7	47.0	35.5	24.7	41.8
LogitBoost by Cai et al. (2006) [36]	56.9	51.5	45.4	30.2	46.0
Logistic regression by Kurgan and Homaeian (2006) [10]	69.1	61.6	60.1	38.3	57.1
SVM by Kurgan and Chen (2007) [37]	77.4	66.4	61.3	45.4	62.7
AATP by Zhang et al. (2012) [31]	81.9	74.7	75.1	55.8	71.7
AAC-PSSM-AC by Liu et al. (2012) [23]	85.3	81.7	73.7	55.3	74.1
MLR model by Xia et al. (2012) [11]	92.6	72.5	71.7	71.0	77.2
MODAS by Mizianty and Kurgan (2009) [33]	92.3	83.7	81.2	68.3	81.4
SVM by Ding et al. (2012) [32]	95.03	81.26	83.24	77.55	84.34
Our method	94.90	95.49	95.83	91.43	94.34

**Table 6:** Performance comparison of different methods on D1185 dataset.

Method	Prediction accuracy (%)				
	All-α	All-β	α/β	α+β	Overall
MLR model by Xia et al. (2012) [11]	95.6	81.0	78.9	71.9	80.1
Our method	83.06	82.68	79.79	88.50	84.61

**Table 7:** Performance comparison of different methods on D8244 dataset.

Method	Prediction accuracy (%)				
	All-α	All-β	α/β	α+β	Overall
MLR model by Xia et al. (2012) [11]	92.0	85.0	83.2	74.4	83.1
Our method	86.98	90.72	93.52	82.12	88.42

**Table 8:** Performance comparison of different methods on Z277 dataset.

Method	Prediction accuracy (%)				
	All-α	All-β	α/β	α+β	Overall
Rough sets by Cao et al.(2006) [20]	77.1	77.0	93.8	66.2	79.4
Information-theoretical approach by Zheng et al. (2010) [22]	87.1	80.3	93.8	67.7	83.0
LogitBoost by Feng et al. (2005) [21]	81.4	88.5	92.6	72.3	84.1
VPMCD by Raghuraj and Lakshminarayanan(2008) [38]	85.7	85.0	92.9	84.4	84.2
IGA-SVM by Li et al. (2008) [24]	84.3	88.5	92.6	70.7	84.5
CWT-PCA-SVM by Li et al. (2009) [25]	85.7	90.2	87.7	80.1	85.9
Markov-SVM by Qin et al. (2012) [27]	90.0	85.2	86.4	81.5	85.9
AAC-PSSM-AC by Liu et al. (2012) [23]	88.6	95.1	97.5	81.5	91.0
Our method	100.00	98.31	100.00	100.00	99.64

**Table 9:** Performance comparison of different methods on Z498 dataset.

Method	Prediction accuracy (%)				
	All-α	All-β	α/β	α+β	Overall
Rough sets by Cao et al.(2006) [20]	87.9	91.3	97.1	86.0	90.8
SVM fusion by Chen et al. (2006) [39]	99.1	96.0	80.9	91.5	91.4
Markov-SVM by Qin et al. (2012) [27]	91.6	94.4	96.3	91.5	93.6
NN-CDM by Liu et al. (2010) [40]	96.3	93.7	95.6	89.9	93.8
Information-theoretical approach by Zheng et al. (2010) [22]	95.3	93.7	97.8	88.3	93.8
IGA-SVM by Li et al. (2008) [24]	96.3	93.6	97.8	89.2	94.2
LogitBoost by Feng et al. (2005) [21]	92.6	96.0	97.1	93.0	94.8
CWT-PCA-SVM by Li et al. (2009) [25]	94.4	96.8	97.0	92.3	95.2
AAC-PSSM-AC by Liu et al. (2012) [23]	94.4	96.8	97.8	93.8	95.8
Our method	100.00	99.15	100.00	100.00	99.79

For two high similarity datasets Z277 and Z498, our method reached the overall accuracies of 99.64% and 99.79% (**Tables 8** **&** **9**), which are still better than the other classifiers including Rough sets [20], LogitBoost [21], Information-theoretical approach [22], AAC-PSSM-AC [23], and SVM-based methods [24], [25]. We noticed that the other approach based on PSSM features, AAC-PSSM-AC, also achieved a very high prediction accuracy. This illustrates that PSI-BLAST profile is indeed a very useful predictor for protein structural class prediction.

Actually, there are still many proteins without known GO annotations and structural classes. Motivated by the observation that similar proteins are prone to share the same GO annotation [26], we here propose a possible solution to this problem, and wish to incorporate it into our future prediction model. Given a new protein without known of GO terms, we first collect all proteins homologous to it in terms of sequence similarity by BLAST, and then use all available GO terms of its homologies to measure the GO features of this query protein. For example, we could simply use the geometrical center of all its homologous GO features to represent this protein.

To highlight the effectiveness of the recursive-based feature selection, we compared it with another commonly used feature selection tool, F-score [5] (**Fig. 3**). As is shown, the prediction accuracies by SVM-RFE are remarkably higher than those by F-score. Taken 1189 dataset as an example, the total accuracy by SVM-RFE strategy is 96.40%, which is 24.93% higher than that by F-score. It shows that the recursive-based feature ranking, which could grasp the combination effects among different features, is superior to individual-based feature selections.

As the case study, we predicted the structural classes of ten proteins, most of them are colorectal cancer-related proteins (**Table 10**). For example, the centrosome (CEP55_HUMAN) is the major microtubule-organizing centre of animal cells and through its influence on the cytoskeleton is involved in cell shape, polarity and motility. It belongs to the all-α folding class and is up-regulated in colon cancer according to our previous research. As is shown in **Table 10**, this protein was consistently predicted as α-helical protein by our predictor on all five training datasets. Another example is the tyrosine-protein kinase receptor UFO (UFO_HUMAN), which is highly expressed in metastatic colon tumors and primary colon tumors. Our predictor training by all five datasets also correctly predicted it as an all-β protein.

**Table 10:** Examples to show the predicted results by our predictor based on five datasets.

Accession Number	Entry name	Structural class	Training dataset				
			1189	D640	25PDB	Z277	Z498
Q53EZ4	CEP55_HUMAN	All-α	All-α	All-α	All-α	All-α	All-α
P30530	UFO_HUMAN	All-β	All-β	All-β	All-β	All-β	All-β
Q9H6I2	SOX17_HUMAN	All-α	All-α	All-α	All-α	All-α	All-α
O60318	GANP_HUMAN	All-α	All-α	All-α	All-α	α+β	All-α
O15105	SMAD7_HUMAN	All-β	All-β	All-β	All-β	All-β	All-β
Q8TD84	DSCL1_HUMAN	All-β	All-β	All-β	All-β	All-β	All-β
P60953	CDC42_HUMAN	α/β	α/β	All-α	α/β	α/β	α/β
Q8F4I0	METX_LEPIN	α/β	α/β	α/β	α/β	α/β	α/β
Q15024	EXOS7_HUMAN	α+β	α+β	α+β	α+β	α+β	α+β
Q8XL08	OGA_CLOPE	α+β	α+β	α+β	α+β	α+β	α+β

## Conclusions

**Figure 5:**
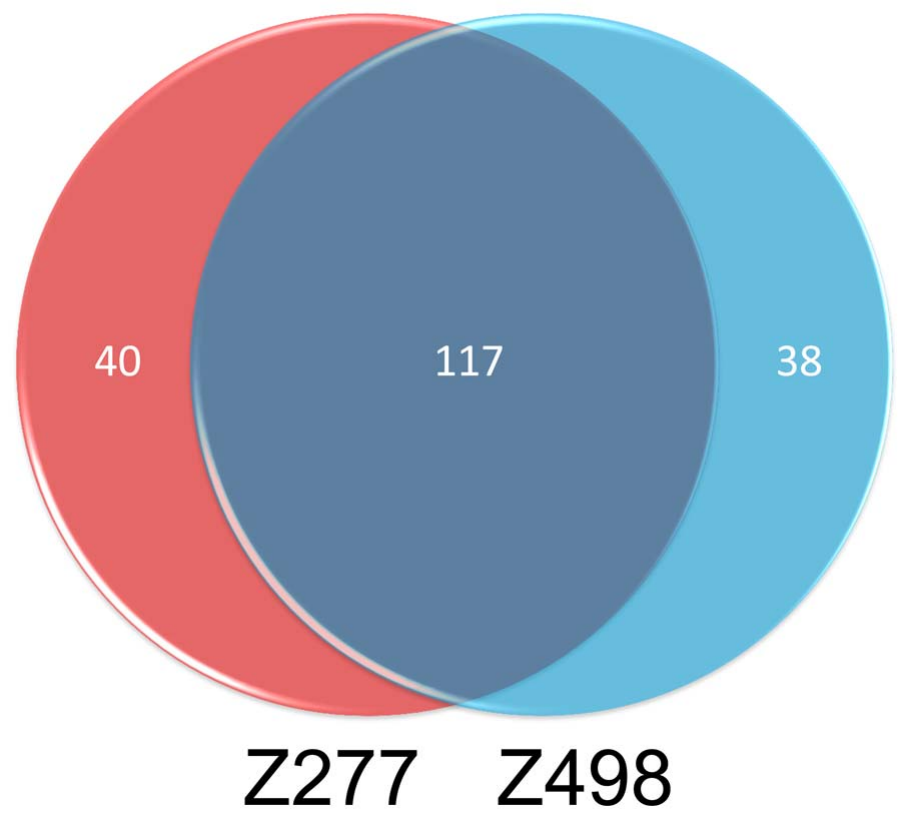
This graph shows the overlapped PROFEAT features of Z277 and Z498. After feature selection by SVM-REF, 157 and 155 PROFEAT features are selected in top322 features for datasets Z277 and Z498, and have significant overlap (117 common features).

In this study, we introduced a recursive feature selection scheme based on linear kernel SVM in order to select the optimal features from three kinds of important features, i.e., protein GO function annotation, amino acid physical-chemical properties and PSI-BLAST profile. Validation tests on seven benchmark datasets show that the selected features are more effective in identifying protein structural classes than those of other feature selection methods. For two high similarity datasets, Z277 and Z498, our prediction accuracies reach 99.64% and 99.79%, which respectively are 8.64% and 3.99% higher than state-of-the-art methods. Moreover, the selected top features are very consistent, in which PROFEAT constitutes the greater part (**Fig. 5**). As for the low similarity datasets, i.e., 1189, D640 and 25PDB, the total accuracies are 96.40%, 95.70% and 94.34%, which are higher than other approaches based on the same datasets. As for our test on datasets D1185 and D8244, high total accuracies of 84.61% and 88.42% were achieved, which are 4.5% and 5.3% higher than those of the predicted secondary structure-based methods.

However, our method suffers from marginally higher computational complexity than the F-score bases for feature ranking methods. Our method may be unable to predict the secondary structural class for a few proteins due to a lack of their GO numbers. Despite these observations, our approach could effectively catch more core features than other feature ranking methods and thus helpful to improve the prediction of protein structural classes. This effectiveness in recognizing classification patterns provides encouragement and support to future studies. We could apply our method to other classification problems. Some examples include protein-binding sites prediction, highly effective antiviral peptides prediction and siRNA efficacy prediction.

## Supporting Information

Figure S1 **The ROC curves for D640 dataset.**
(TIF)

Figure S2 **The ROC curves for 25PDB dataset.**
(TIF)

Figure S3 **The ROC curves for Z277 dataset.**
(TIF)

Figure S4 **The ROC curves for Z498 dataset.**
(TIF)

Figure S5 **The ROC curves for D1185 dataset.**
(TIF)

Figure S6 **The ROC curves for D8244 dataset.**
(TIF)

Datasets S1 **Seven datasets used in this study.**
(RAR)
