# Supplementary figures and images for: PSSP-RFE: Accurate Prediction of Protein Structural Class by Recursive Feature Extraction from PSI-BLAST Profile, Physical-Chemical Property and Functional Annotations
Source: PLoS One. 2014 Mar 27;9(3):e92863. doi: 10.1371/journal.pone.0092863 (PMC3968047; doi:10.1371/journal.pone.0092863)

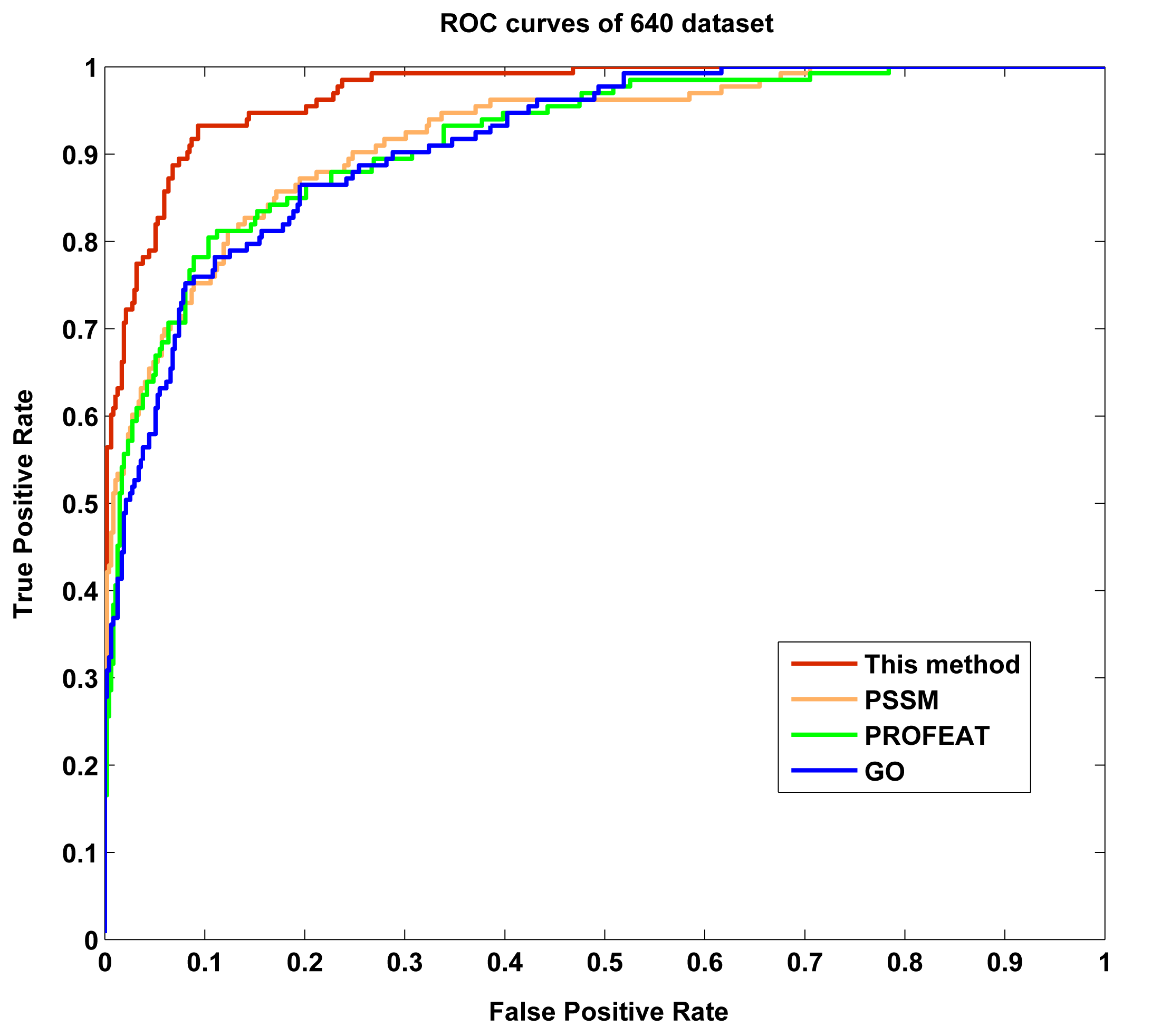

Supplement: Figure S1 — The ROC curves for D640 dataset. (TIF) [file pone.0092863.s001.tif]

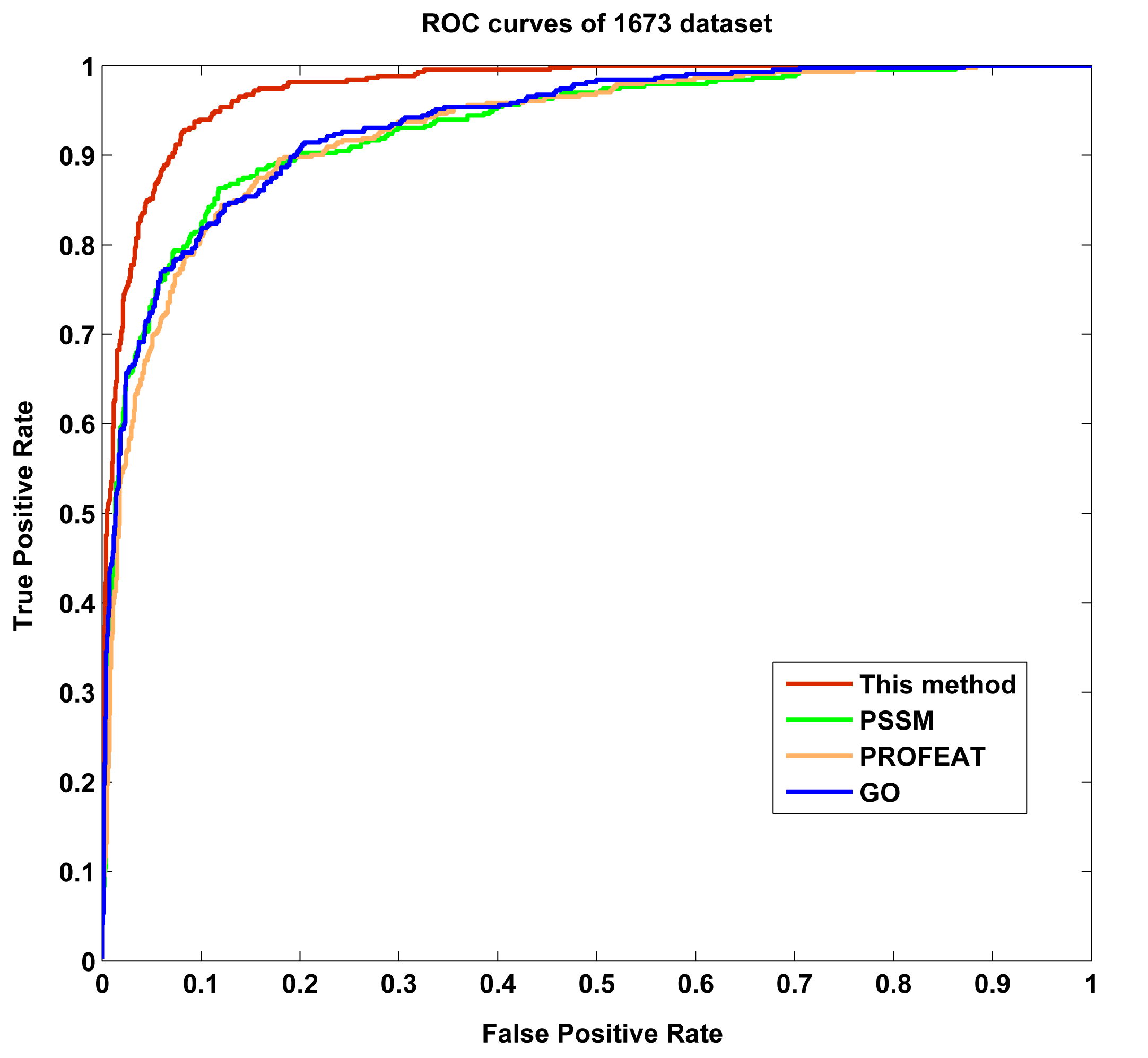

Supplement: Figure S2 — The ROC curves for 25PDB dataset. (TIF) [file pone.0092863.s002.tif]

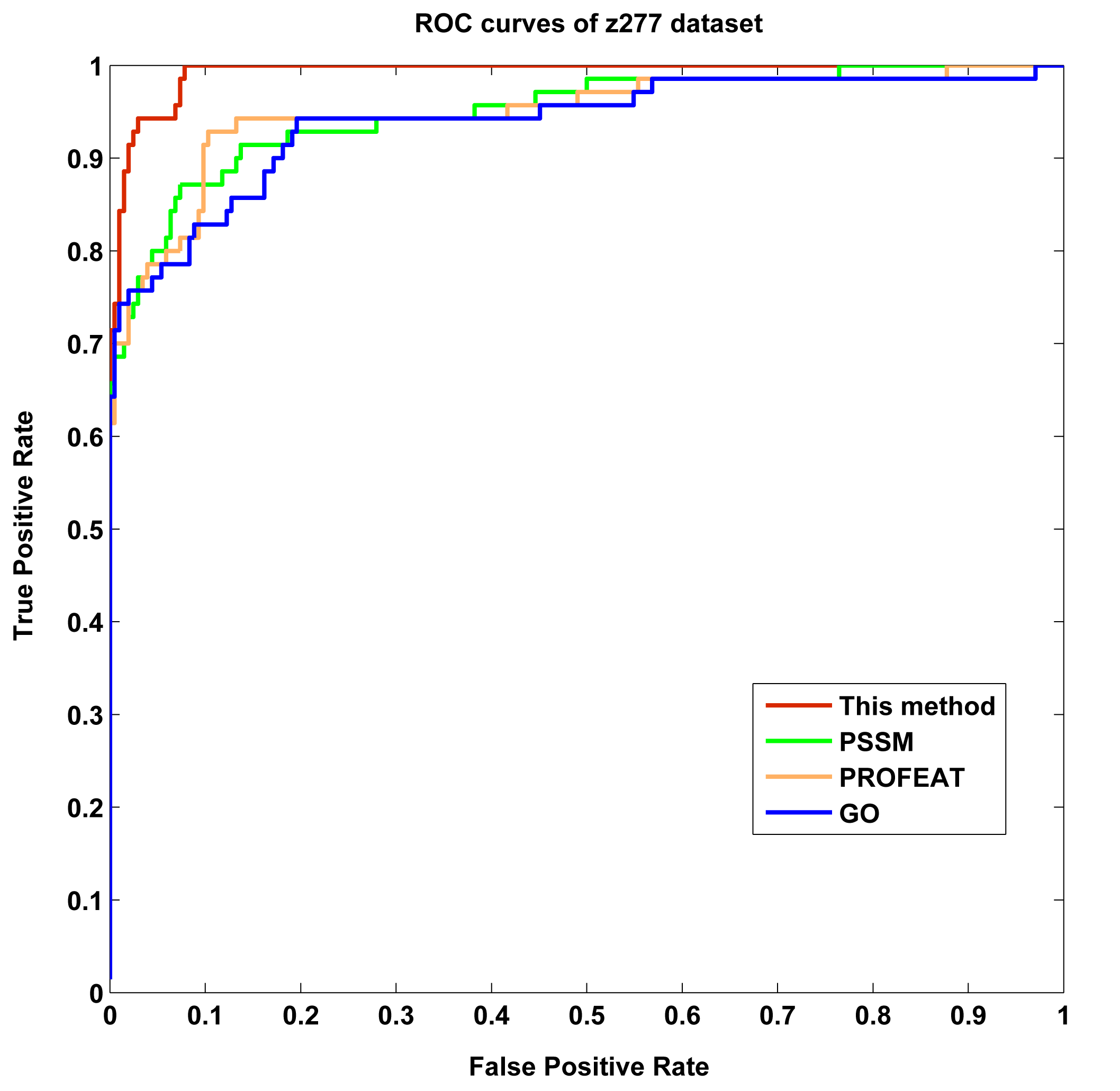

Supplement: Figure S3 — The ROC curves for Z277 dataset. (TIF) [file pone.0092863.s003.tif]

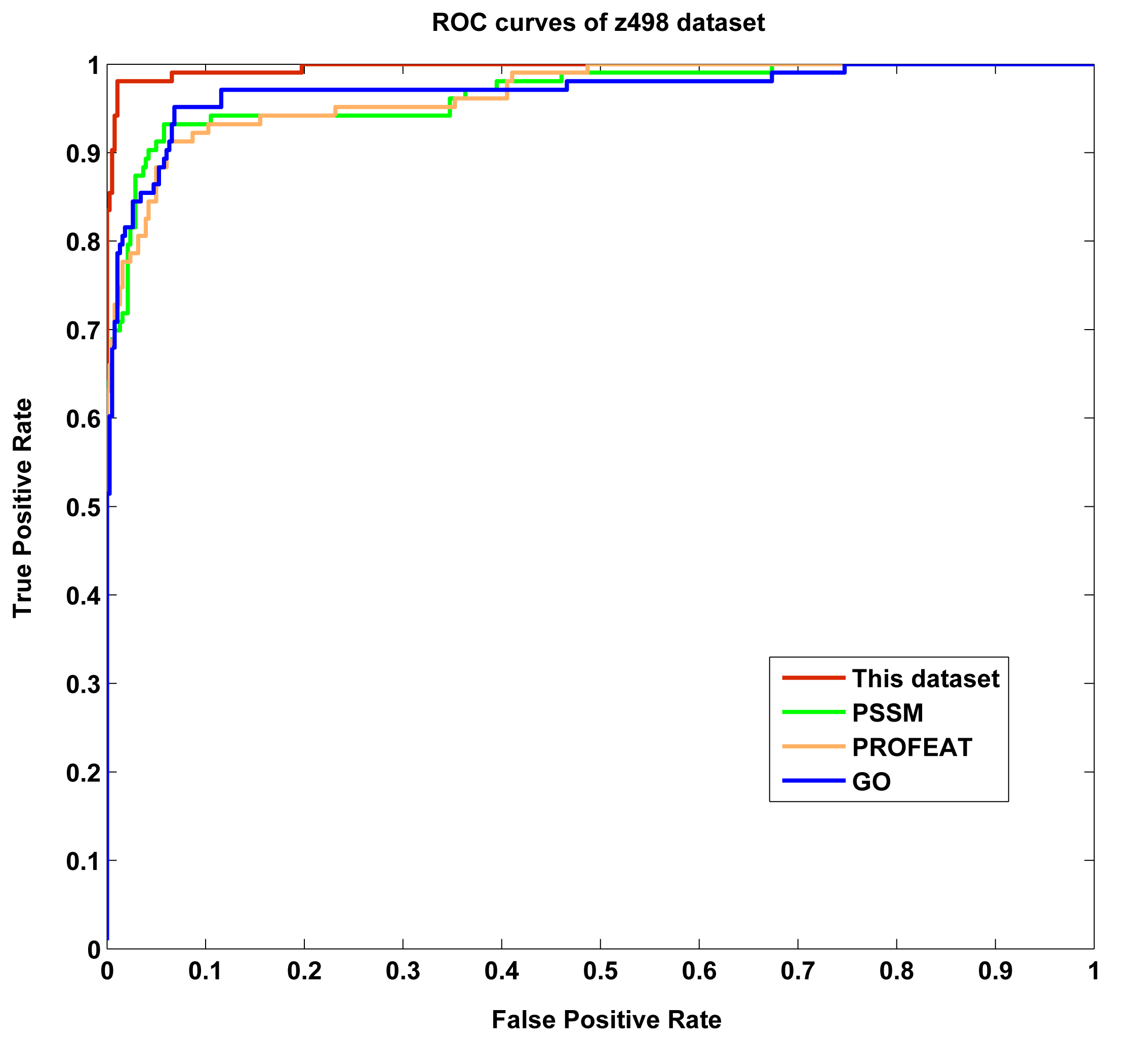

Supplement: Figure S4 — The ROC curves for Z498 dataset. (TIF) [file pone.0092863.s004.tif]

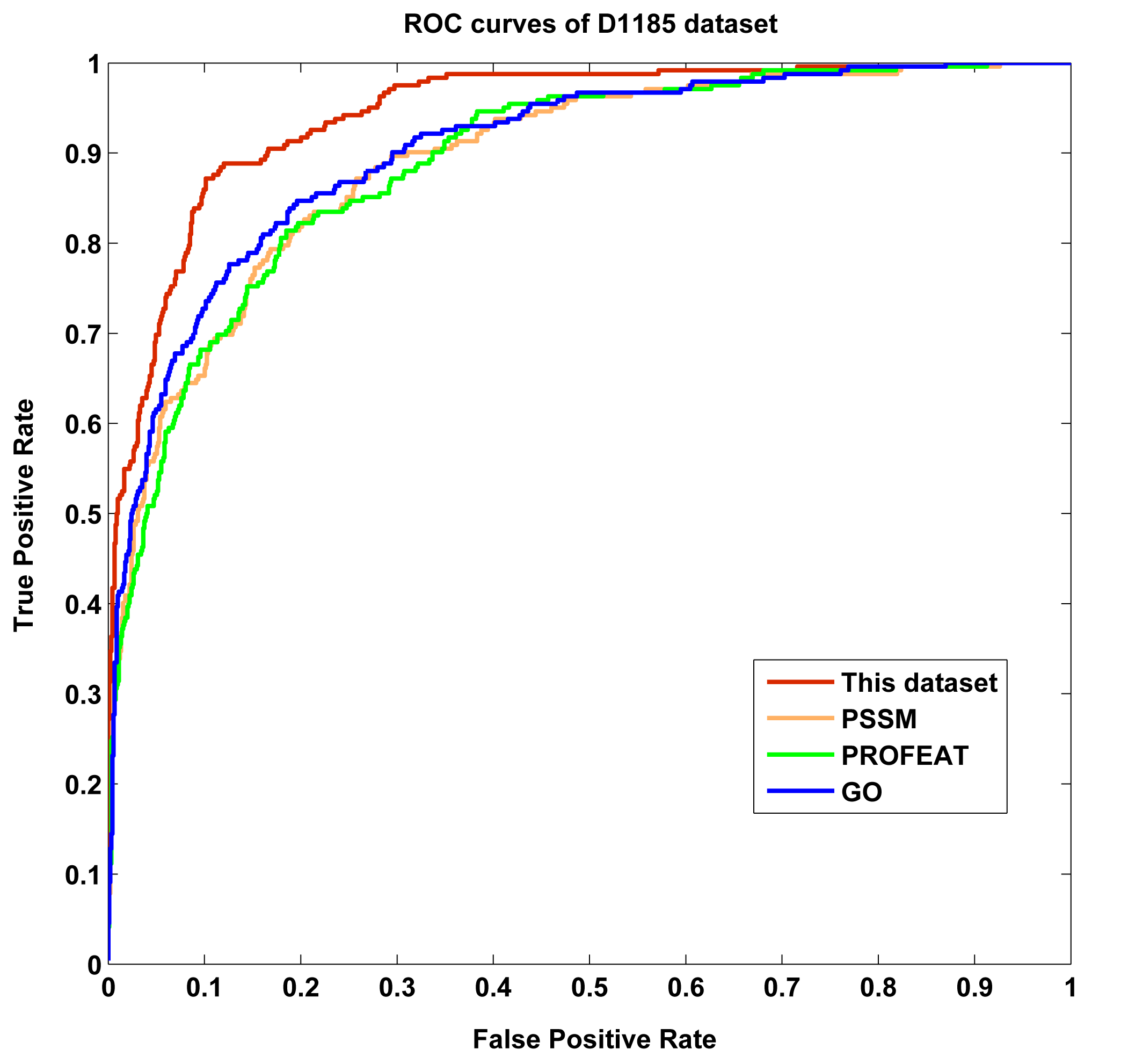

Supplement: Figure S5 — The ROC curves for D1185 dataset. (TIF) [file pone.0092863.s005.tif]

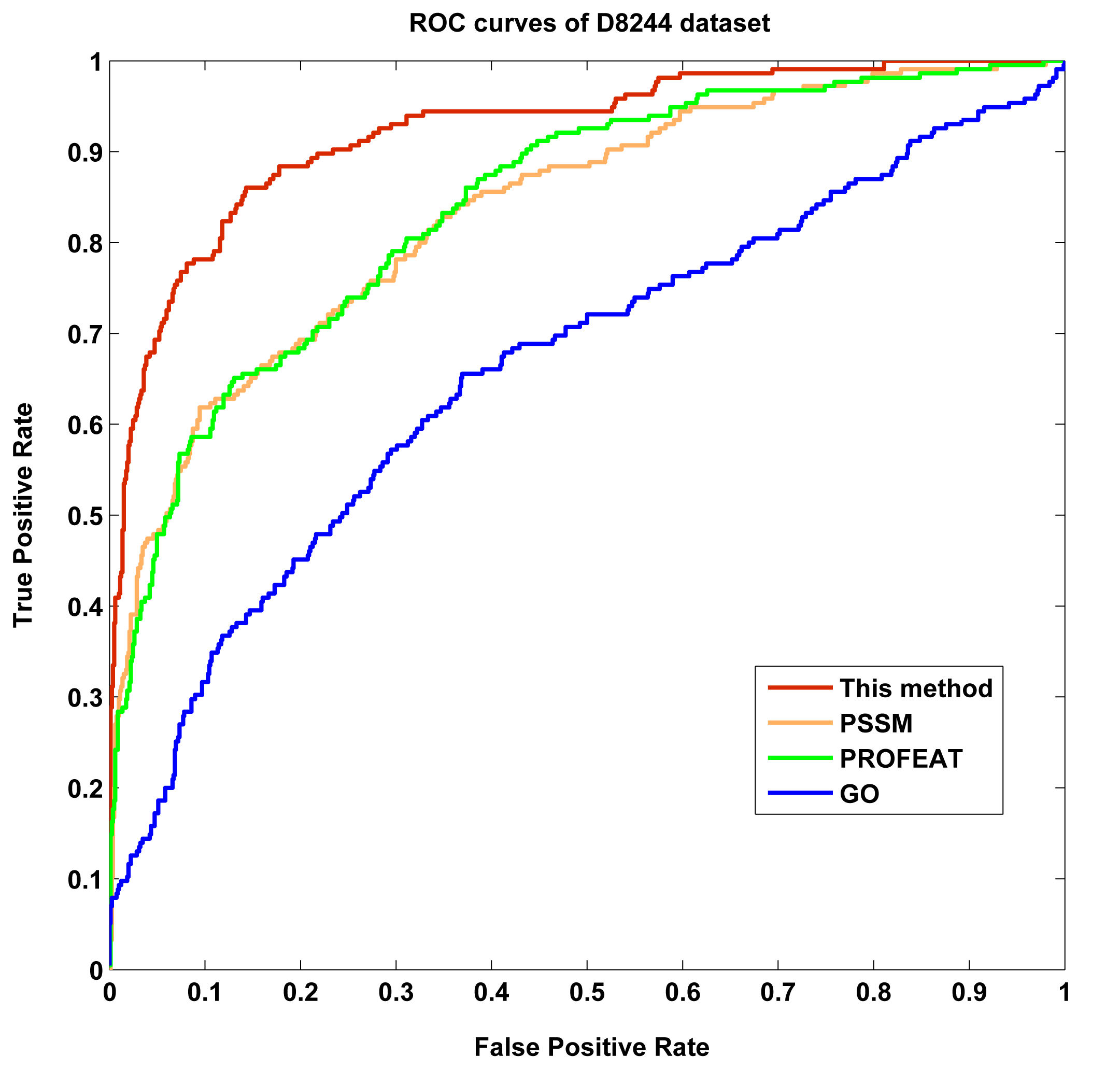

Supplement: Figure S6 — The ROC curves for D8244 dataset. (TIF) [file pone.0092863.s006.tif]
